# Comparison of the Anti-Prion Mechanism of Four Different Anti-Prion Compounds, Anti-PrP Monoclonal Antibody 44B1, Pentosan Polysulfate, Chlorpromazine, and U18666A, in Prion-Infected Mouse Neuroblastoma Cells

**DOI:** 10.1371/journal.pone.0106516

**Published:** 2014-09-02

**Authors:** Takeshi Yamasaki, Akio Suzuki, Rie Hasebe, Motohiro Horiuchi

**Affiliations:** Laboratory of Veterinary Hygiene, Graduate School of Veterinary Medicine, Hokkaido University, Kita-ku, Sapporo, Japan; University of Maryland School of Medicine, United States of America

## Abstract

Molecules that inhibit the formation of an abnormal isoform of prion protein (PrP^Sc^) in prion-infected cells are candidate therapeutic agents for prion diseases. Understanding how these molecules inhibit PrP^Sc^ formation provides logical basis for proper evaluation of their therapeutic potential. In this study, we extensively analyzed the effects of the anti-PrP monoclonal antibody (mAb) 44B1, pentosan polysulfate (PPS), chlorpromazine (CPZ) and U18666A on the intracellular dynamics of a cellular isoform of prion protein (PrP^C^) and PrP^Sc^ in prion-infected mouse neuroblastoma cells to re-evaluate the effects of those agents. MAb 44B1 and PPS rapidly reduced PrP^Sc^ levels without altering intracellular distribution of PrP^Sc^. PPS did not change the distribution and levels of PrP^C^, whereas mAb 44B1 appeared to inhibit the trafficking of cell surface PrP^C^ to organelles in the endocytic-recycling pathway that are thought to be one of the sites for PrP^Sc^ formation. In contrast, CPZ and U18666A initiated the redistribution of PrP^Sc^ from organelles in the endocytic-recycling pathway to late endosomes/lysosomes without apparent changes in the distribution of PrP^C^. The inhibition of lysosomal function by monensin or bafilomycin A1 after the occurrence of PrP^Sc^ redistribution by CPZ or U18666A partly antagonized PrP^Sc^ degradation, suggesting that the transfer of PrP^Sc^ to late endosomes/lysosomes, possibly via alteration of the membrane trafficking machinery of cells, leads to PrP^Sc^ degradation. This study revealed that precise analysis of the intracellular dynamics of PrP^C^ and PrP^Sc^ provides important information for understanding the mechanism of anti-prion agents.

## Introduction

Prion diseases are neurodegenerative disorders of mammals that include scrapie in sheep, bovine spongiform encephalopathy (BSE), chronic wasting disease (CWD) in Cervidae, and Creutzfeldt–Jakob disease (CJD) in humans [1]. In prion diseases, an abnormal isoform of prion protein (PrP^Sc^) accumulates in the central nervous system (CNS). PrP^Sc^ is a major component of prion, the causative agent of prion diseases, and generated by conversion of a cellular isoform of prion protein (PrP^C^) encoded by the host *Prnp* gene. The generation and accumulation of PrP^Sc^ in CNS play a crucial role in the progression of pathogenesis. Prion diseases have long incubation periods ranging from months to years; however, once clinical signs appear, the diseases are subacutely progressive and invariably fatal.

There is a great desire for the establishment of therapeutics for prion diseases. Various experimental approaches including pharmacotherapy, immunotherapy, and cell-therapy have been reported. One of the major targets of the therapeutics is believed to be the inhibition of PrP^Sc^ formation or acceleration of PrP^Sc^ degradation, although protection of neurons from neurotoxic conditions and/or regeneration of damaged neurons are also therapeutic target [2]–[4]. To date, numerous compounds have been reported to inhibit PrP^Sc^ formation in cells persistently infected with prions, and a few of them showed prolonged survival time in mouse models, particularly treatments that were initiated in the middle or late stages of prion infection [5]. Moreover, clinical trials of some compounds such as pentosan polysulfate (PPS), doxycycline, and quinacrine, which have been reported to inhibit PrP^Sc^ formation in vivo and in vitro, have been conducted in patients with human prion diseases. However, no compounds have shown significant improvement in survival or clinical features in humans [6]–[10].

The logical basis of the effect of anti-prion compounds are important in the development of pharmacotherapy for prion diseases. Many compounds, such as sulfated glycans, polyanions, polyene antibiotics, tricyclic or tetracyclic compounds, PrP peptides, small interfering RNAs and anti-PrP antibodies, have been shown to prevent PrP^Sc^ formation by blocking the interaction between PrP^C^ and PrP^Sc^, possibly by direct binding to either PrP^C^ or PrP^Sc^, by interference of accessory molecules required for the interaction, by reduction of PrP^C^ expression or by alteration of PrP^C^ distribution [11]. The inhibitors of cholesterol synthesis such as lovastatin, squalestatin, and U18666A are also considered to interfere with PrP^Sc^ formation by changing the distribution of either PrP^C^ or PrP^Sc^ via alteration of cholesterol metabolism [12]–[14]. In contrast, cationic polyamines [15] and autophagy inducers such as lithium, trehalose, FK506, and tamoxifen are reported to eliminate PrP^Sc^ from cells by enhancing the degradation of PrP^Sc^ [16]–[19].

Although the preceding reports have shown the effects of anti-prion compounds on PrP^Sc^ formation, the precise cellular mechanisms of the inhibition of PrP^Sc^ formation remain to be elucidated. Clarification of the intracellular dynamics of PrP^C^ and PrP^Sc^ in prion-infected cells treated with the compounds aids the understanding of precise anti-prion mechanisms. In this study, we established a method that can simultaneously detect PrP^C^ and PrP^Sc^ in an immunofluorescence assay (IFA) by modifying a previously reported PrP^Sc^-specific staining method [20]. Using this method, we compared the effects of four anti-prion compounds, anti-PrP antibody, PPS, chlorpromazine (CPZ), and U18666A, focusing on the kinetics of PrP^Sc^ formation and intracellular dynamics of PrP^C^ and PrP^Sc^.

## Materials and Methods

### Antibodies and regents

Anti-PrP mouse monoclonal antibodies (mAbs) 31C6 and 132 were used to detect PrP [21]. MAb 44B1, which is known to reduce PrP^Sc^ levels in prion-infected cells, was used as one of the anti-prion compounds [22]. Anti-Lamp1 rat mAb (Beckman Coulter, 1D4B), anti-sorting nexin 1 (Snx1) rabbit polyclonal antibodies (Proteintech Group, 10304-1-AP), anti-LC3 rabbit polyclonal antibodies (Medical & Biological Laboratories Co., Ltd, PM036), and anti-early endosome antigen 1 (EEA1) rabbit mAb (Cell Signaling Technology, C45B10) were used for IFA. Anti-β-actin mAb (Sigma, AC-15), anti-cathepsin D rabbit polyclonal antibodies (BioVision, 3191R-100) and anti-GAPDH rabbit polyclonal antibodies (Millipore, ABS16) were used for immunoblotting or dot-blotting. Alexa Fluor 488-conjugated goat F(ab′)_2_ fragment anti-mouse IgG, Alexa Fluor 488- and 555-conjugated goat F(ab′)_2_ fragment anti-rabbit IgG and Alexa Fluor 555 conjugated goat IgG anti-rat IgG (Life Technologies) were used as secondary antibodies for IFA. Alexa Fluor 555-labeled mAb 31C6 (31C6-Af555) was prepared using the Alexa Fluor 555 Monoclonal Antibody Labeling Kit (Life Technologies) according to the manufacturer’s instructions. Fab fragments of mAb 31C6 genetically conjugated with human placental alkaline phosphatase (31C6Fab-PLAP, A.S. and M.H., unpublished) were used for the direct detection of PrP by immunoblotting and dot-blotting. MAb 132 genetically conjugated with enhanced green fluorescent protein (EGFP) at the C-terminus of the heavy chain (rIgG132-EGFP, A.S. and M.H., unpublished) was used for the direct immunostaining of PrP^Sc^ by IFA. Anti-β-actin mAb conjugated with peroxidase by using Peroxidase Labeling Kit-NH_2_ (Dojindo Molecular Technologies) was used for the direct detection of β-actin by immunoblotting. PPS was purchased from Dainippon Sumitomo Pharma. CPZ, U18666A, monensin (Mon), and bafilomycin A1 (BafA1) were purchased from Sigma-Aldrich. Alexa Fluor 488-conjugated acetylated low-density lipoprotein (LDL) was purchased from Life Technologies.

### Cell culture

N2a-3 cells, a subclone of the mouse neuroblastoma cell line Neuro2a [23], and N2a-3 cells persistently infected with the 22L prion strain (ScN2a-3-22L [24]) were used.

### Treatment of cells with anti-prion compounds

N2a-3 cells or ScN2a-3-22L cells were plated in 6- or 12-well plates (Thermo Scientific) or on 8-well Lab-Tek II chambered coverglass (Thermo Scientific) at a 1∶10 ratio. The cells were cultured in Dulbecco’s modified Eagle’s medium (DMEM; ICN Biomedicals) containing 10% fetal bovine serum (FBS; Gibco), 1% non-essential amino acids (NEAAs; Life Technologies), and penicillin (100 U/ml) streptomycin (100 µg/ml) (Life Technologies) at 37°C in a 5% CO_2_ atmosphere for 24 h in 6- or 12-well plates or for 48 h on 8-well Lab-Tek II chambered coverglass. Subsequently, the culture medium was replaced with DMEM containing 10% FBS, 1% NEAAs and each anti-prion compound. The cells were cultured up to 72 h in the presence of each anti-prion compound, and then subjected to immunoblotting, dot-blotting or IFA.

### Immunoblotting

Cells grown on 6- or 12-well plate were lysed in lysis buffer [23]. Preparation of samples to monitor proteinase K (PK)-resistant PrP^Sc^ (PrP-res) or other molecules, SDS-PAGE and immunoblotting were performed as previously described [23], [24].

### Dot-blotting

To monitor PrP^C^, PrP-res or GAPDH, the cell lysate equivalent to 40 µg of total protein was transferred onto a polyvinylidene difluoride (PVDF) membrane using a dot-blotter (Bio-Rad). To detect PrP-res, the PVDF membrane was treated with 20 µg/ml PK for 1 h at 37°C and subsequently incubated with 1 mM Pefabloc SC for 15 min at 4°C. The membrane was treated with 50 µg/ml DNase I for 15 min at room temperature (rt) and subsequently incubated in 3 M guanidine thiocyanate (GdnSCN) for 30 min at rt. For the direct immunodetection of PrP, the PVDF membrane was incubated with 31C6Fab-PLAP (42 ng/ml) in 1% skim milk-PBS containing 0.1% Tween 20 (PBST) at 4°C overnight. The chemiluminescence detection was conducted using CDP-Star (Applied Biosystems) according to the manufacturer’s instructions. For the detection of GAPDH, the PVDF membrane was incubated with anti-GAPDH rabbit polyclonal antibodies (2 µg/ml) in 1% skim milk-PBST at rt for 2 h followed by an incubation with anti-rabbit IgG Horseradish Peroxidase F(ab’)_2_ fragment (GE Healthcare). The chemiluminescence detection was carried out as described [23], [24].

### IFA

IFA was performed as previously described [25]. For the direct staining of PrP^Sc^, cells were incubated with rIgG132-EGFP (2 µg/ml) at 4°C overnight. For the double staining of PrP^C^ and PrP^Sc^, cells treated with 4% paraformaldehyde and 0.1% Triton X-100 were blocked with 5% FBS and incubated with 31C6-Af555 (2 µg/ml) at 4°C overnight. The cells were washed with PBS and subsequently fixed again with 4% paraformaldehyde in PBS for 10 min. The cells were treated with 5 M GdnSCN for 10 min and subsequently incubated with rIgG132-EGFP (2 µg/ml) at 4°C overnight. Nuclei were counterstained with 5 µg/ml 4′,6-diamidino-2-phenylindole, dilactate (DAPI; Life Technologies) in PBS at rt for 30 min. Finally, the media chamber was filled with PBS and confocal fluorescent images were acquired with a ×63 objective lens on a Zeiss LSM700 inverted microscope and ZEN 2009 software. Z-series of the images were taken at 0.8-µm steps from the top to the bottom of the cells in the area.

### Co-localization statistics

Quantitative co-localization analysis of PrP^Sc^ with organelle markers was performed as previously described [25]. The co-localization ratio that represents a percentage of the voxels of PrP^Sc^ signals co-localized with signals of each organelle marker relative to the total voxels of PrP^Sc^ signals was quantified using the Coloc module in IMARIS software (Bitplane).

### Measurement of PrP^Sc^ or LDL fluorescence intensity in Lamp1-positive vesicles

Acquired Z-series of the multichannel images were reconstructed to a three-dimensional image by the IMARIS software. The isosurface models of Lamp1-positive vesicles were created by the Surpass module in the IMARIS software and the signals of PrP^Sc^ or LDL in the isosurface of Lamp1-positive vesicles were extracted. The intensity of the signals of PrP^Sc^ or LDL per cell were calculated by dividing the total intensities of the signals by the total number of cells in the view field.

## Results

### Effect of anti-prion compounds on the amount of PrP^Sc^

We chose four anti-prion compounds, mAb 44B1, PPS, CPZ and U18666A. The mAb 44B1 is reported to inhibit the internalization of PrP^C^ [22], and PPS is reported to inhibit the binding of PrP^C^ to PrP^Sc^ or alter the intracellular trafficking of PrP^C^ and/or PrP^Sc^ [26]. CPZ is a derivative of a tricyclic compound which causes the redistribution of cholesterol from the plasma membrane to intracellular compartments [14]. U18666A interferes with the intracellular trafficking involved in the recycling of cholesterol between the plasma membrane and intracellular compartments [27], [28].

**Figure 1:**
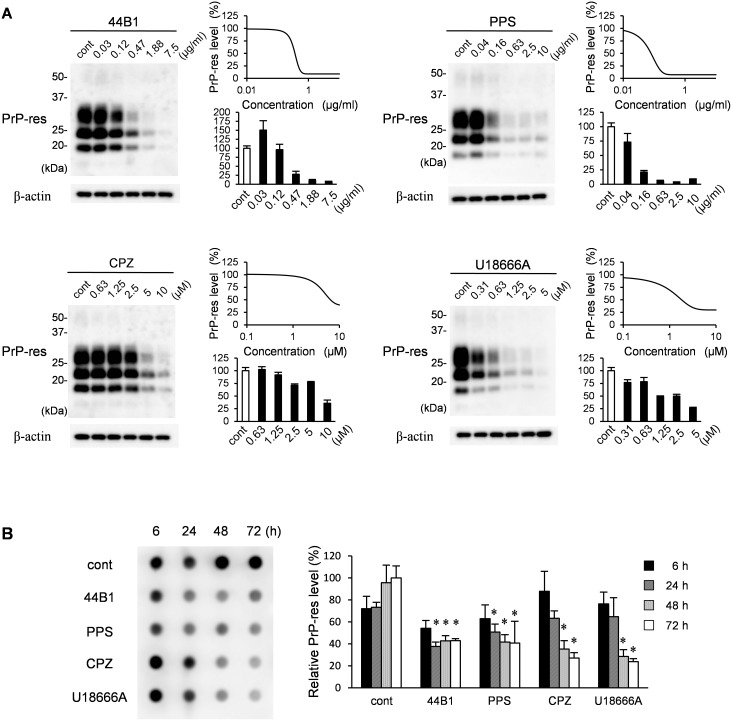
Influence of anti-prion compounds on the amount of PrP-res. (A) ScN2a-3-22L cells grown on 12-well plates were cultured in the presence or absence of mAb 44B1, PPS, CPZ, or U18666A at the indicated concentration for 72 h. The samples were subjected to immunoblotting and dot-blotting for PrP-res detection or β-actin detection for endogenous control. Representative blots for each compound are shown on the left. The graph on the right shows PrP-res levels relative to the control samples. The means and standard deviations (SDs) of four independent experiments (PrP-res was detected by dot-blotting) are indicated. Graphs on the upper right show the logistic curve fitted to the data of PrP-res levels by dot-blotting (B) ScN2a-3-22L cells were cultured with anti-prion compounds at the EC_65_ (mAb 44B1, 0.4 µg/ml; PPS, 0.1 µg/ml; CPZ, 10 µM; U18666A, 5 µM) for the indicated time and subjected to dot-blotting for PrP-res. Representative dot-blotting is shown on the left, and the graph on the right shows the levels of PrP-res relative to the samples from ScN2a-3-22L cells cultured without anti-prion compounds for 72 h. The means and SDs of four independent experiments are depicted. Asterisks indicate a significant difference compared with the control at the same time point (Student’s *t-*test, *p*<0.05).

First, we re-evaluated the effect of these compounds on the formation of PrP-res in ScN2a-3-22L cells, which are persistently infected with the 22L prion strain. All compounds were confirmed to reduce PrP-res levels in ScN2a-3-22L cells in a concentration-dependent manner after 72 h of treatment (Fig. 1A). Next, we analyzed the kinetics of PrP-res levels in cells treated with each compound at an effective concentration that decreased PrP-res levels by 65% after 72 h of treatment (EC_65_ was estimated by concentration-effect curves in Fig. 1A). Compared with the mock-treated control at each time point, mAb 44B1 or PPS significantly decreased PrP-res levels 24 h after the initiation of the treatment (Fig. 1B). In contrast, significant decrease in PrP-res levels were observed after 48 h of CPZ or U189666A treatment (*p*<0.05). During 72 h of the treatments, any of the compounds did not show apparent adverse effects on cell viability: the cell viabilities went up during first 48 h-treatment and then reached plateau levels (Fig. S1). This result suggests that the reduction of PrP-res was not due to the cytotoxic effects of the compounds.

### Effect of anti-prion compounds on the localization of PrP^Sc^

**Figure 2:**
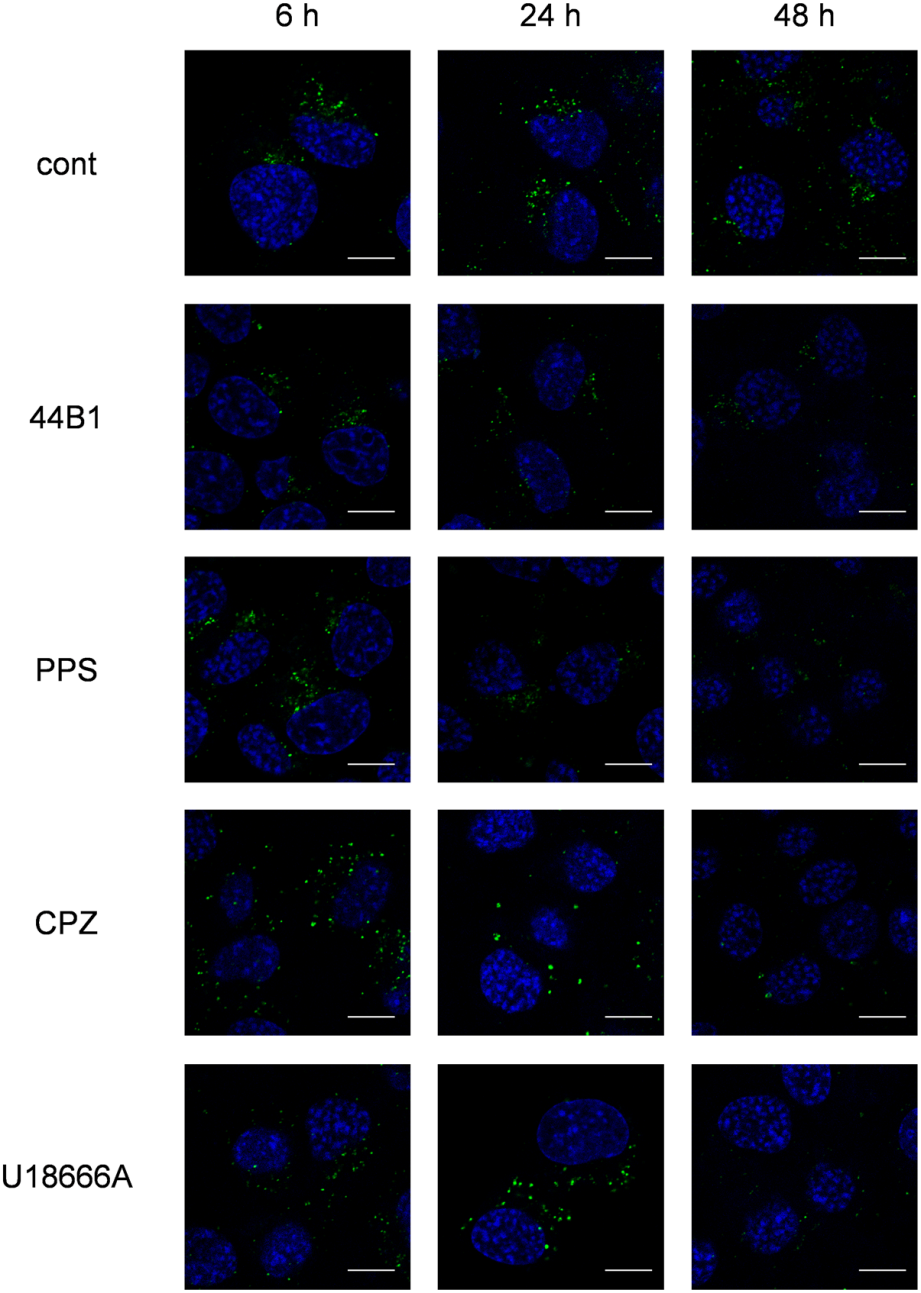
Influence of anti-prion compounds on the intracellular PrP^Sc^ distribution. ScN2a-3-22L cells grown on a chambered coverglass for 48 h were cultured with or without an anti-prion compound at the EC_65_ (mAb 44B1, 0.4 µg/ml; PPS, 0.1 µg/ml; CPZ, 10 µM; U18666A, 5 µM) for 6, 24, or 48 h. The cells were subjected to PrP^Sc^-specific detection by direct immunostaining with rIgG132-EGFP. The cell nuclei were counterstained with DAPI. The panels show the merged images of PrP^Sc^ (green) and nuclei (blue). Scale bars: 10 µm.

To elucidate the intracellular events involved in the reduction of PrP^Sc^, we analyzed the intracellular localization of PrP^Sc^ in ScN2a-3-22L cells treated with the compounds for up to 48 h. To avoid the reaction of secondary antibodies to mAb 44B1, direct immunostaining of PrP^Sc^ was performed using the EGFP-tagged mAb 132 (rIgG132-EGFP). As previously reported [20], PrP^Sc^ in mock-treated cells mainly clustered in peri-nuclear regions of the cells (Fig. 2). Treatment with mAb 44B1 or PPS did not change the localization of PrP^Sc^, but it reduced the signal intensity of PrP^Sc^ by 24 h (Fig. 2). In contrast, PrP^Sc^ in cells treated with CPZ or U18666A appeared to be dispersed throughout cytoplasm after 6 h of treatment (Fig. 2). Moreover, larger granular staining of PrP^Sc^ was observed in the cytoplasm after 24 h of CPZ or U18666A treatment, and the intensities of the granular PrP^Sc^ were decreased after an additional 24 h (Fig. 2).

### Effect of anti-prion compounds on PrP^C^ metabolism

**Figure 3:**
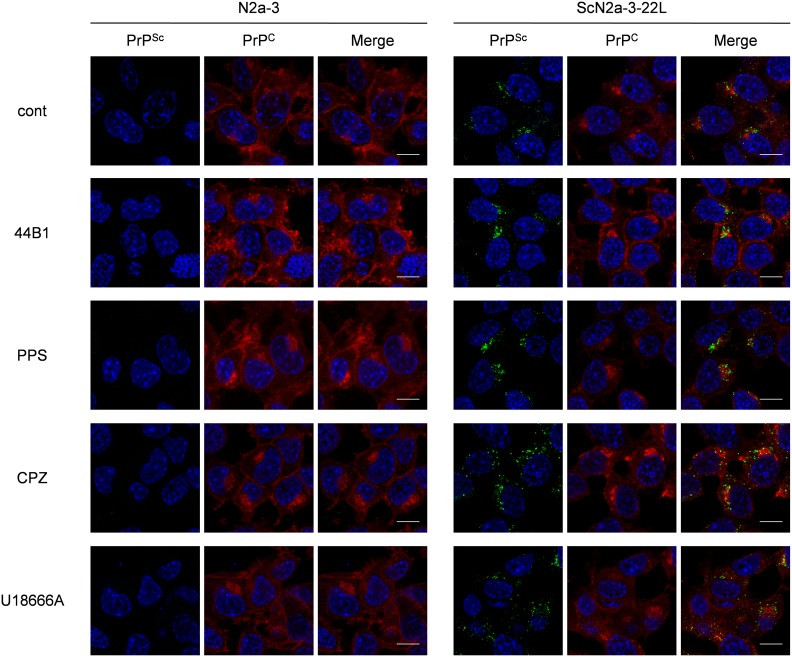
Influence of anti-prion compounds on the localization of PrP^Sc^ and PrP^C^. N2a-3 cells or ScN2a-3-22L cells were cultured with or without an anti-prion compound at the EC_65_ (mAb 44B1, 0.4 µg/ml; PPS, 0.1 µg/ml; CPZ, 10 µM; U18666A, 5 µM) for 6 h. The cells were fixed and stained with 31C6-Af555 to detect PrP^C^, and subsequently subjected to PrP^Sc^-specific detection with rIgG132-EGFP. The cell nuclei were counterstained with DAPI. The merged images of PrP^Sc^ (green) and nuclei (blue) are shown on the left, those of PrP^C^ (red) and nuclei are shown in the middle, and those of PrP^Sc^, PrP^C^, and nuclei are shown on the right. Scale bars: 10 µm.

Next, we analyzed the distributions of PrP^C^ in ScN2a-3-22L cells treated with the anti-prion compounds. For the simultaneous staining of PrP^C^ and PrP^Sc^, mAb 31C6 conjugated with Alexa Fluor 555 (31C6-Af555) was first incubated with fixed ScN2a-3-22L cells to stain PrP^C^. After staining with 31C6-Af555, the cells were fixed again and treated with 5 M GdnSCN for PrP^Sc^-specific staining with rIgG132-EGFP. Only a few signals of PrP stained with 31C6-Af555 were co-localized with PrP^Sc^ signals stained with rIgG132-EGFP in ScN2a-3-22L cells (Fig. S2), suggesting that most of the signals stained with 31C6-Af555 were derived from PrP^C^ and, if any, PrP^Sc^ that cannot be detected using rIgG132-EGFP. No positive signals from un-infected N2a-3 cells, but granular staining from ScN2a-3-22L cells with rIgG132-EGFP demonstrated the PrP^Sc^-specific staining with rIgG132-EGFP (Fig. 3).

In mAb 44B1-treated ScN2a-3-22L cells, intense PrP^C^ staining at the plasma membrane was observed compared with the findings in mock-treated control cells, whereas PPS treatment did not appear to change the distribution of PrP^C^ after 6 h (Fig. 3). The distribution of PrP^C^ in cells treated with CPZ or U18666A was also unchanged compared with the control cells, although PrP^Sc^ clustering at the peri-nuclear regions was less obvious but PrP^Sc^ appeared to be widely distributed in the cytoplasm (Fig. 3). Quantification of PrP^C^ signals revealed that the signal intensities of PrP^C^ per cells were not changed in PPS, CPZ or U18666A-treated cells compared with mock-treated control cells, whereas the intensities increased 3-fold in mAb 44B1-treated cells (data not shown).

**Figure 4:**
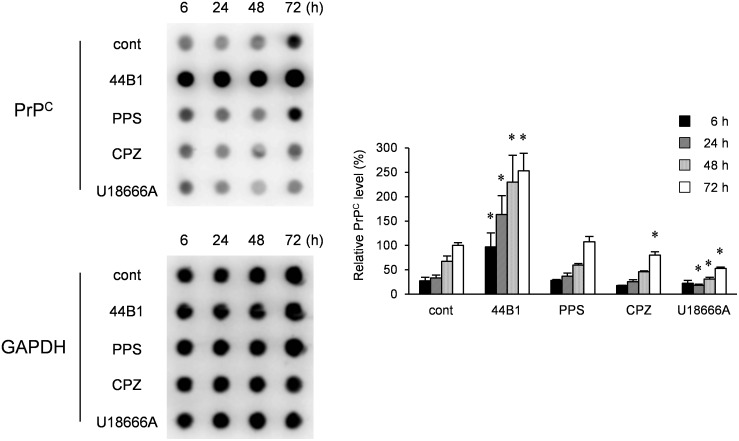
Influence of anti-prion compounds on PrP^C^ levels. N2a-3 cells were cultured with an anti-prion compound at the EC_65_ (mAb 44B1, 0.4 µg/ml; PPS, 0.1 µg/ml; CPZ, 10 µM; U18666A, 5 µM) for 6–72 h and subjected to dot-blotting for the detection of PrP^C^ or GAPDH for endogenous control. Representative dot-blot images are shown on the left, and the graph on the right shows the PrP^C^ levels relative to that of 72-h mock-treated cells. The means and SDs of three independent experiments are depicted. Asterisks indicate a significant difference between the cells treated with each anti-prion compound and mock-treated control cells at the same time point (Student’s *t-*test, *p*<0.05).

Next, we analyzed the effect of long-term exposure to these compounds on PrP^C^ levels using un-infected N2a-3 cells. The levels of PrP^C^ in control N2a-3 cells increased in a time-dependent manner (Fig. 4), which was consistent with cell density-dependent increases of PrP^C^ levels [24]. In the cells treated with mAb 44B1, the amount of PrP^C^ was markedly increased compared to that in the control cells during the incubation periods. In contrast, PrP^C^ levels in CPZ- or U18666A-treated cells were significantly lower than that in mock-treated cells at 72 h and 24–72 h after treatment, respectively. PPS did not change PrP^C^ levels if cells were treated at EC_65_ (Fig. 4). These results suggest that long exposure (>24 h) of cells to CPZ or U18666A affects PrP^C^ metabolism.

### CPZ and U18666A treatments induce redistribution of PrP^Sc^ from early endosomes/endocytic recycling compartments (ERCs) to late endosomes/lysosomes

**Figure 5:**
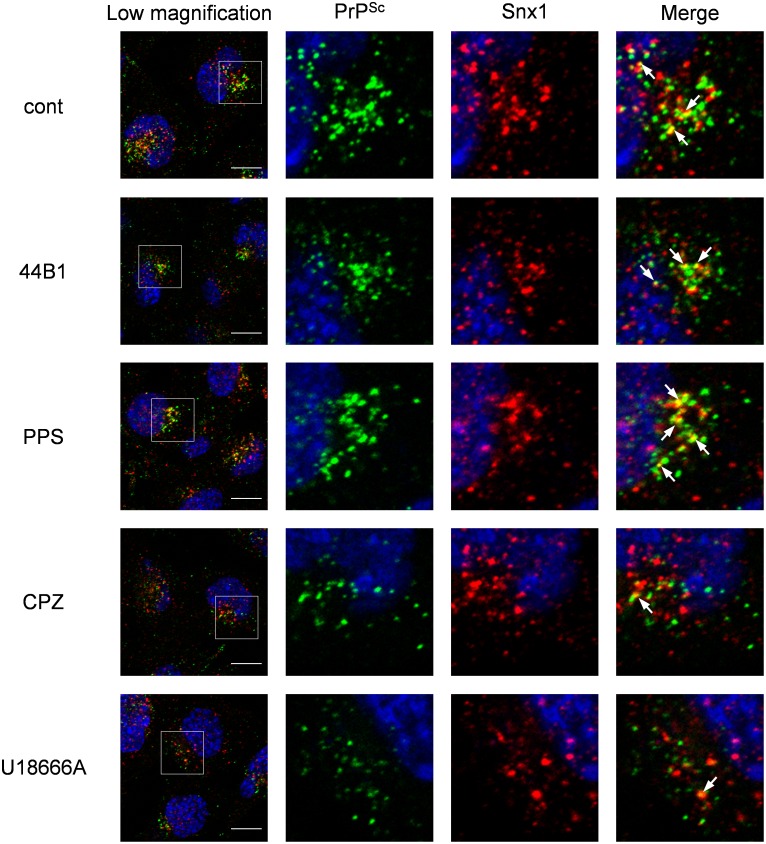
Co-localization of PrP^Sc^ with Snx1. ScN2a-3-22L cells grown on a chambered coverglass for 48 h were incubated with 7.5 µg/ml mAb 44B1, 10 µg/ml PPS, 10 µM CPZ, or 5 µM U18666A or without an anti-prion compound for 2 h. The cells were subjected to PrP^Sc^-specific staining with rIgG132-EGFP and immunostaining for Snx1. Nuclei were counterstained with DAPI. The leftmost column presents a lower-magnification merged image of PrP^Sc^ (green), Snx1 (red), and nuclei (blue). Individual and merged high-magnification images of the boxed regions are shown on the right. Arrows denote representative examples of co-localization of PrP^Sc^ with Snx1. Scale bars: 10 µm.

Next, we analyzed the distribution of PrP^Sc^ in cells treated with anti-prion compounds. For this experiment, we employed the highest concentration of anti-prion compounds used in Figure 1 to obtain a maximum inhibitory effect on the formation of PrP^Sc^. Double-staining of PrP^Sc^ and Snx1, which is a component of retromers involved in retrograde transport from early endosomes to the *trans*-Golgi network [29], revealed that the localization of PrP^Sc^ in CPZ- or U18666A-treated cells was altered within 2 h after the initiation of the treatment; compared with the co-localization of PrP^Sc^ with Snx1 in the mock-, mAb 44B1-, or PPS-treated cells, the co-localization was less obvious in CPZ- or U18666A-treated cells (Fig. 5). These results suggest that the change in the PrP^Sc^ distribution occurred in a short period after the start of CPZ or U18666A treatment.

**Figure 6:**
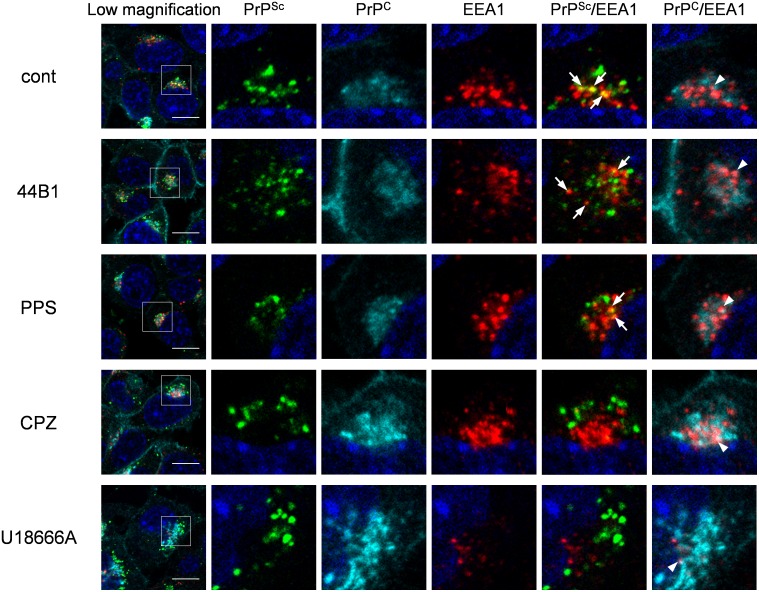
Co-localization of PrP^Sc^ or PrP^C^ with EEA1. ScN2a-3-22L cells were cultured with 7.5 µg/ml mAb 44B1, 10 µg/ml PPS, 10 µM CPZ or 5 µM U18666A or without an anti-prion compound for 6 h. The cells were subjected to direct immunostaining of PrP^C^ and PrP^Sc^ with 31C6-Af555 and rIgG132-EGFP, respectively and subsequently to immunostaining for EEA1 and nuclei. The leftmost column shows a lower-magnification merged image of PrP^Sc^ (green), PrP^C^ (cyan), EEA1 (red), and nuclei (blue). Individual and merged high-magnification images of the boxed regions are shown on the right. Arrows or arrowheads denote representative examples of the co-localization of PrP^Sc^ with EEA1 or PrP^C^ with EEA1, respectively. Scale bars: 10 µm.

**Figure 7:**
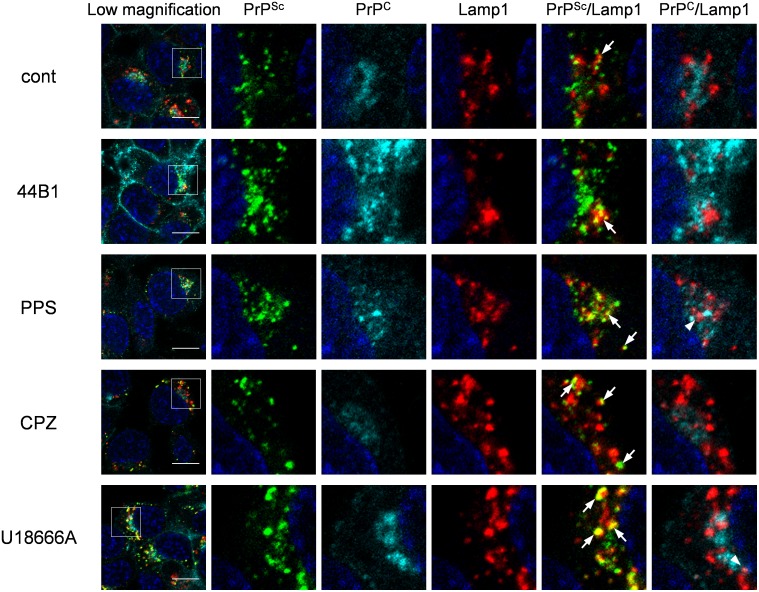
Co-localization of PrP^Sc^ or PrP^C^ with Lamp1. ScN2a-3-22L cells were cultured under the same condition as described in Figure 6. The cells were subjected to direct immunostaining of PrP^C^ and PrP^Sc^ with 31C6-Af555 and rIgG132-EGFP, respectively and subsequently to immunostaining for Lamp1 and nuclei. The leftmost column shows a lower-magnification merged image of PrP^Sc^ (green), PrP^C^ (cyan), Lamp1 (red), and nuclei (blue). Individual and merged high-magnification images of the boxed regions are shown on the right. Arrows or arrowheads denote representative examples of the co-localization of PrP^Sc^ with Lamp1 or PrP^C^ with Lamp1, respectively. Scale bars: 10 µm.

**Figure 8:**
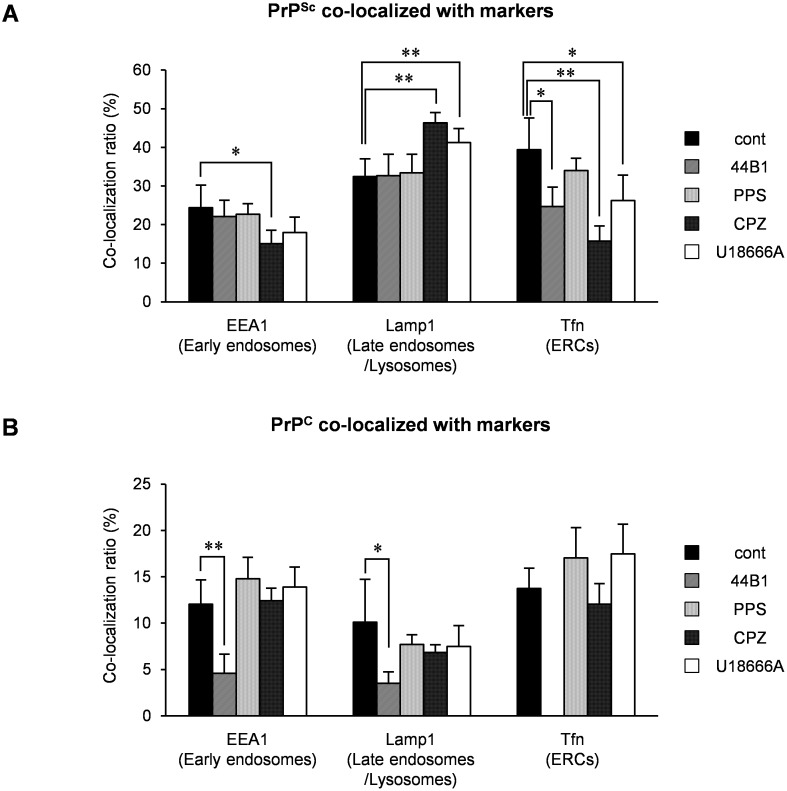
Co-localization statistics of PrP^Sc^ or PrP^C^ with organelle markers. Co-localization analyses of the images shown in Figures 7 and 8 and Figure S3 were conducted. (A) Ratio of double-positive areas for PrP^Sc^ and markers to the sum of PrP^Sc^-positive areas. (B) Ratio of double-positive areas for PrP^C^ and markers to the sum of PrP^C^ positive areas. The means and SDs of the value acquired in five or six view fields are shown. Single (*p*<0.05) and double (*p*<0.01) asterisks indicate a significant difference compared with the control.

We previously reported that PrP^Sc^ exists throughout endocytic compartments including early endosomes and ERCs, which are organelles in the endocytic-recycling pathway that are involved in the recycling of lipids or membrane proteins, and late endosomes and lysosomes, which are organelles in the endo-lysosomal pathway that are involved in the degradation of intracellular or exogenously introduced molecules [20]. Therefore, we further analyzed the localization of both PrP^Sc^ and PrP^C^ in cells treated with the anti-prion compounds using the following endosome markers: EEA1 for early endosomes, exogenously introduced transferrin (Tfn) for ERCs, and Lamp1 for late endosomes/lysosomes. Similar to the co-localization of PrP^Sc^ with endosome markers in mock-treated cells, some PrP^Sc^ granular staining was co-localized with EEA1 (Fig. 6), Lamp1 (Fig. 7), and Tfn (Fig. S3) in ScN2a-3-22L cells treated with mAb 44B1 or PPS. In contrast, PrP^Sc^ granular staining in ScN2a-3-22L cells treated with CPZ or U18666A did not appear to be co-localized well with EEA1 (Fig. 6) and Tfn (Fig. S3), but the staining was co-localized with Lamp1 (Fig. 7) after 6 h of the treatment. Quantitative analysis of the co-localization indicated that the co-localization ratio of PrP^Sc^ with EEA1 or Tfn in cells treated with CPZ was significantly lower than that in the mock-treated cells (Fig. 8A). On the other hand, the co-localization ratio of PrP^Sc^ with Lamp1 was significantly increased (Fig. 8A). Likewise, a decrease in the co-localization of PrP^Sc^ with EEA1 (not statistically significant) or Tfn (*p*<0.05) and an increase in the co-localization of PrP^Sc^ with Lamp1 (*p*<0.01) were observed in cells treated with U18666A (Fig. 8A). In contrast, there were no significant differences in the co-localization ratios of PrP^C^ with EEA1, Lamp1, and Tfn in cells treated with PPS, CPZ, or U18666A compared with the findings in the mock-treated cells (Fig. 8B). Interestingly, the co-localization ratio of PrP^C^ with EEA1 or Lamp1 was significantly decreased in cells treated with mAb 44B1 (Fig. 8B). These results suggest that CPZ and U18666A induce the redistribution of PrP^Sc^ from EEA1-positive early endosomes and/or Tfn-positive ERCs to Lamp1-positive late endosomes/lysosomes without remarkably affecting in the distribution of PrP^C^.

**Figure 9:**
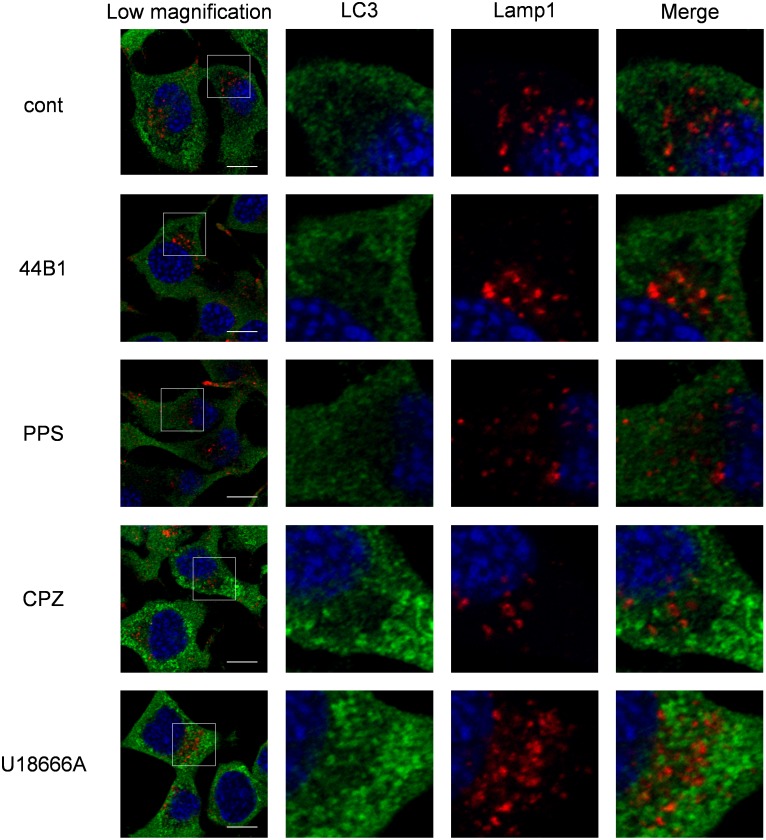
Induction of autophagy by CPZ or U18666A treatment. ScN2a-3-22L cells were cultured under the same conditions as described in Figure 6. The cells were subjected to immunostaining for LC3 and Lamp1 and counterstained with DAPI. The leftmost column shows the lower-magnification merged image of LC3 (green), Lamp1 (red), and nuclei (blue). Individual and merged high-magnification images of the boxed regions are shown on the right. Scale bars: 10 µm.

Lysosomes can fuse with different cellular membranes, such as endosomes, autophagosomes, phagosomes and the plasma membrane [30]. In the process of macroautophagy, autophagosomes that contain cytosolic constituents fuse with lysosomes to form autolysosomes for degrading internal materials [31]. To assess whether autophagy is involved in the redistribution of PrP^Sc^ induced by CPZ or U18666A treatment, we analyzed the expression of the autophagosome marker LC3. LC3 accumulation was observed in the cytoplasm of the cells treated with CPZ or U18666A (Fig. 9); however, LC3 did not co-localize with Lamp1. This indicates the absence of the formation of autolysosome.

### CPZ or U18666A treatment causes the degradation of PrP^Sc^ in secondary lysosomes

**Figure 10:**
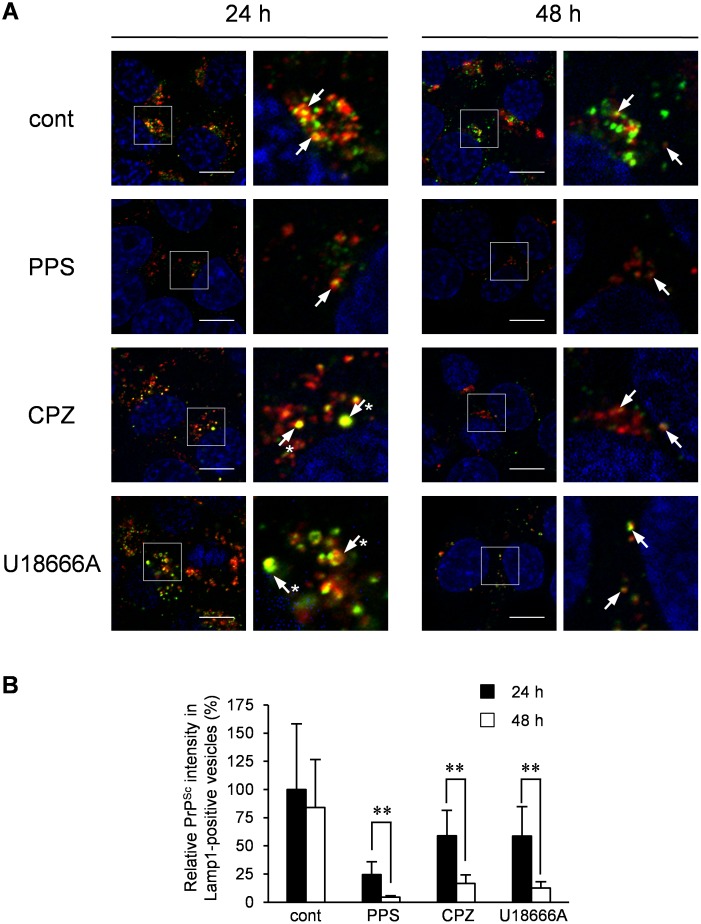
Fate of PrP^Sc^ after treatment with CPZ or U18666A. ScN2a-3-22L cells were cultured with 10 µg/ml PPS, 10 µM CPZ or 5 µM U18666A or without an anti-prion compound for 24 or 48 h. The cells were subjected to the PrP^Sc^-specific indirect immunostaining of PrP^Sc^ with mAb 132 and immunostaining for Lamp1 and nuclei. (A) Localization of PrP^Sc^. The left columns show a lower-magnification merged image of PrP^Sc^ (green), Lamp1 (red), and nuclei (blue). The high-magnification images of the boxed regions are shown on the right. Arrows indicate representative co-localization of PrP^Sc^ with Lamp1. Arrows with asterisks indicate swollen Lamp1-positive vesicles positive for PrP^Sc^. Scale bars: 10 µm. (B) Intensity of PrP^Sc^ in Lamp1-positive vesicles. The graph represents the values of the fluorescent intensities of PrP^Sc^ in Lamp1-positive vesicles per cell relative to those of PrP^Sc^ in Lamp1-positive vesicles per cell in mock-treated cells after 24 h of treatment. The means and SDs of the value acquired in six view fields are shown. Double asterisks indicate a significant difference between cells treated for 24 and 48 h (Student’s *t-*test, *p*<0.01).

**Figure 11:**
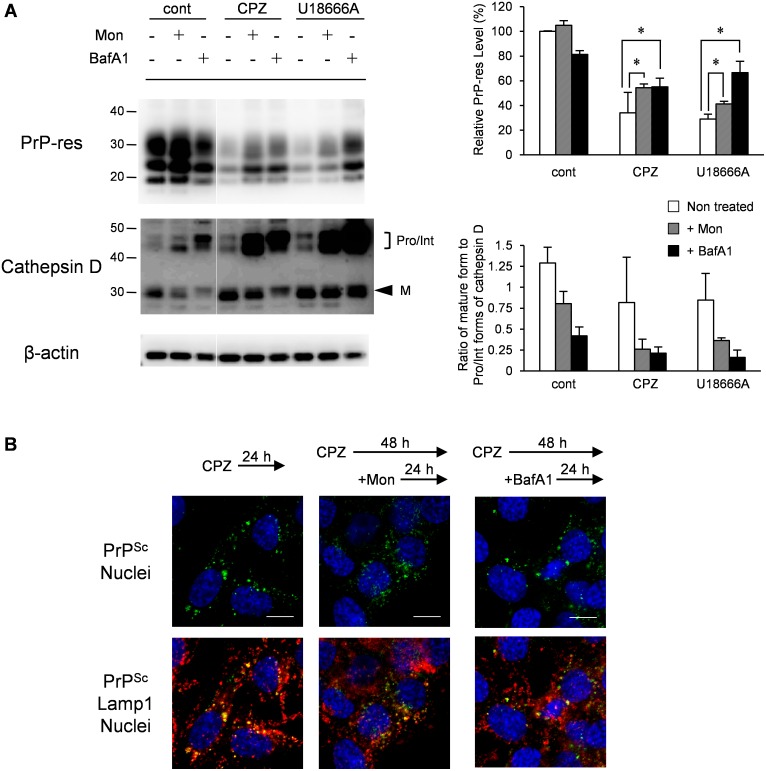
Effect of lysosomal hydrolysis inhibition on the decrease of PrP^Sc^ levels induced by CPZ or U18666A treatment. (A) Immunoblot analysis of PrP-res and cathepsin D. ScN2a-3-22L cells were cultured with 10 µM CPZ or 5 µM U18666A or without compounds for 24 h. Subsequently, monensin (Mon) or bafilomycin A1 (BafA1) was added to the culture at a final concentration of 100 or 5 nM, respectively. Following an additional incubation for 36 h with or without Mon or BafA1, the cells were subjected to immunoblotting for PrP-res, cathepsin D, or β-actin. Representative immunoblot images are shown on the left. The bracket in the immunoblot of cathepsin D denotes the pro- and/or intermediate forms of cathepsin D (Pro/Int). The arrowhead denotes the mature form of cathepsin D (M). The upper right graph shows the levels of PrP-res relative to the control. The lower right graph shows the ratio of mature to pro−/intermediate forms of cathepsin D. The means and SDs of three independent experiments are depicted. Asterisks indicate a significant difference between Mon- or BafA1-treated samples and untreated samples (non-treated) (Student’s *t-*test, *p*<0.05). (B) Localization of PrP^Sc^. ScN2a-3-22L cells grown on a chambered coverglass were cultured with 10 µM CPZ for 24 h. Subsequently, Mon or BafA1 was added at a final concentration of 100 or 5 nM, respectively, and the cells were cultured for additional 24 h in the presence of CPZ and Mon or BafA1. The cells were subjected to double-staining of PrP^Sc^ and Lamp1 before (left) and after the treatment with Mon (middle) or BafA1 (right). The upper panel shows the merged images of PrP^Sc^ (green) and nuclei (blue). The bottom panel shows the merged images of PrP^Sc^ (green), Lamp1 (red) and nuclei (blue). Scale bars: 10 µm.

To analyze the fate of PrP^Sc^ transported to Lamp1-positive late endosomes/lysosomes upon CPZ or U18666A treatment, PrP^Sc^ in Lamp1-positive vesicles was monitored for 48 h after the initiation of treatment. After 24 h of CPZ or U18666A treatment, a large portion of PrP^Sc^ existed in swollen Lamp1-positive vesicles (Fig. 10A, 24 h, arrows with asterisks). After an additional 24 h of incubation, swollen Lamp1-positive vesicles disappeared, but PrP^Sc^ remained in Lamp1-positive vesicles, although its intensity was significantly decreased (Figs. 10A and B, 48 h). To evaluate whether the decrease in PrP^Sc^ levels was because of the degradation of PrP^Sc^ in Lamp1-positive vesicles, we analyzed PrP^Sc^ after impairing lysosomal hydrolysis. For this purpose, we treated the cells with Mon, a Na^+^/H^+^-exchanging ionophore [32], or BafA1, which is known as an inhibitor of vacuolar H^+^-ATPase [33], at 24 h after the start of CPZ or U18666A treatment, at which point a large portion of PrP^Sc^ was transported to Lamp1-positive vesicles. In cells treated with Mon or BafA1, the ratio of the mature form of cathepsin D to pro- and/or intermediate forms of cathepsin D was decreased (Fig. 11A, cathepsin D), suggesting that lysosomal hydrolysis was partly impaired by interfering with the maturation of lysosomal hydrolases. Treatment of cells with Mon or BafA1 partly interfered with the reduction of PrP-res levels that was caused by CPZ or U18666A treatment (Fig. 11A, PrP-res). In IFA, granular staining of PrP^Sc^ in Lamp1-positive vesicles, which was induced by CPZ treatment, was observed after 24 h of treatment with Mon or BafA1 (Fig. 11B). The degradation of PrP^Sc^ induced by CPZ was partly inhibited by the impairment of lysosomal hydrolysis, indicating that CPZ and possibly U18666A treatment causes the degradation of PrP^Sc^ in Lamp1-positive lysosomes.

**Figure 12:**
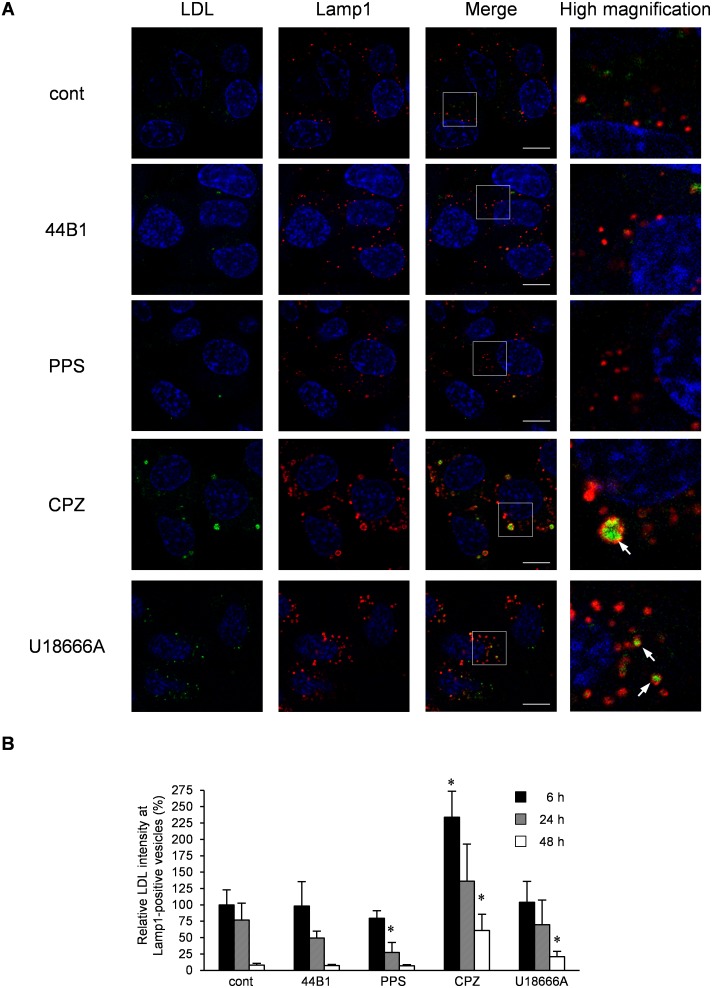
Degradation of LDL in cells treated with anti-prion compounds. ScN2a-3-22L cells were incubated with Alexa Fluor 488-conjugated LDL (4 µg/ml) for 6 h. After the incubation, the cells were cultured in the presence or absence of 0.4 µg/ml mAb 44B1, 0.1 µg/ml PPS, 10 µg/ml CPZ, or 5 µM U18666A for 6–48 h. The cells were subjected to immunostaining of Lamp1 and staining of the cell nuclei with DAPI. Z-series of the images were acquired at 0.8-µm steps from the top to the bottom of the cells in the area. (A) Localization of LDL. The panel shows the representative images of the signals of LDL (green), Lamp1 (red) and nuclei (blue) in cells treated with the indicated anti-prion compound for 24 h. The merged images of LDL and nuclei are shown on the left, those of Lamp1 and nuclei are shown in the middle, and those of LDL, Lamp1, and nuclei are shown on the right. The rightmost column presents the higher-magnification images of the boxed regions in the second right column. Scale bars: 10 µm. (B) Intensity of LDL in Lamp1-positive vesicles. The graph shows the values of the fluorescent intensities of LDL in Lamp1-positive vesicles per cell relative to those of LDL in Lamp1-positive vesicles per cell in mock-treated control cells for 6 h. The means and SDs of the value acquired in five view fields are shown. Asterisks indicate a significant difference compared with the control at the same time point (Student’s *t-*test, *p*<0.05).

The reduction of PrP-res by CPZ or U18666A treatment was observed 48 h after the initiation of treatment (Figs. 1–2), although PrP^Sc^ was already redistributed to Lamp1-positive vesicles within 6 h (Fig. 7). In contrast, PrP-res was rapidly decreased by mAb 44B1 or PPS treatment within 24 h (Fig. 1B). To clarify the difference between the treatments, we analyzed the function of lysosomal hydrolysis in the cells using LDL. LDL binds to the LDL receptor, after which it is internalized from the cell surface via clathrin-coated pits and transported to late endocytic compartments for degradation [34]. Therefore, we measured the degradation of exogenously introduced Alexa Fluor 488-conjugated LDL to monitor the function of lysosomal hydrolysis. After 24 h of treatment, the exogenously introduced LDL was located in the swollen Lamp1-positive vesicles in the cells treated with CPZ or U18666A, whereas LDL was hardly detected in Lamp1-positive vesicles in cells treated with mAb 44B1 or PPS (Fig. 12A). These facts suggest that hydrolysis in the swollen Lamp1-positive vesicles was partly impaired by CPZ or U18666A. The quantification of the intensities of LDL in Lamp1-positive vesicles revealed that the degradation of LDL was significantly inhibited in CPZ-treated cells after 6 h and 48 h of treatment (Fig. 12B, CPZ) or in U18666A-treated cells after 48 h of treatment compared with the findings in mock-treated cells (Fig. 12B, U18666A), whereas significant inhibition of the degradation of LDL was not observed in mAb 44B1- or PPS-treated cells (Fig. 12B, 44B1 and PPS). These results suggest that lysosomal hydrolysis in swollen Lamp1-positive vesicles in CPZ- and U18666A-treated cells was partially impaired and that this impairment accounts for the slow decrease in PrP^Sc^ levels in CPZ- and U186666A- treated cells.

## Discussion

In this study, we re-evaluated four anti-prion compounds and found that mAb 44B1 and PPS rapidly decrease PrP^Sc^ levels, whereas, CPZ and U18666A slowly decrease PrP^Sc^ levels. Detailed analyses of the distribution of PrP^C^ and PrP^Sc^ revealed differences in the mechanism of the inhibition of PrP^Sc^ formation for the four anti-prion compounds.

MAb 44B1 reduced PrP-res levels within 24 h (Fig. 1) without altering the distribution of PrP^Sc^ (Fig. 2). Consistent with a previous report, increase in PrP^C^ at the plasma membrane indicated that mAb 44B1 retained PrP^C^ on the cell surface as an antigen–antibody complex [22]. This idea is further supported by decreased co-localization of PrP^C^ with markers for early or late endosomes (Fig. 8). Therefore, the major anti-prion effect of mAb 44B1 is explained by interference with the internalization and trafficking of PrP^C^ to endocytic compartments where the conversion of PrP^C^ to PrP^Sc^ occurs [25], [28]. The difference in the anti-prion effects of PPS and mAb 44B1 was that PPS decreased PrP-res levels without altering the distribution or amount of PrP^C^, whereas mAb 44B1 did not (Figs. 4 and 8). Although it is reported that PPS stimulates the internalization of PrP^C^ and redistribution of PrP^C^ into late endosomes and/or lysosomes in N2a cells overexpressing chicken PrP^C^ [26] or decreases PrP^C^ levels at higher concentrations (e.g., 100 µg/ml) [35], PPS did not decrease PrP^C^ levels at the EC_65_ (Fig. 4) and did not appear to alter the intracellular localization of PrP^C^ even at 10 µg/ml, a concentration 125-fold than the EC_50_ for PPS in this study (0.08 µg/ml) (Figs. 6–8). These results were supported by the report that PPS treatment does not influence the normal metabolism of PrP^C^ in prion-infected mouse neuroblastoma cells [36]. Although the precise mechanism by which PPS treatment reduces PrP^Sc^ levels could not be identified in this study, PPS may competitively interfere with the binding of PrP^C^ and PrP^Sc^ via endogenous GAG [36]; alternatively, PPS causes the fragmentation of PrP^Sc^ at the cell surface as observed with a heparin-mimetic compound [37].

Interestingly, in contrast to mAb 44B1 and PPS, CPZ and U18666A treatment induced the redistribution of PrP^Sc^ to Lamp1-positive vesicles prior to the alteration of PrP^C^ distribution or PrP^C^ levels (Figs. 3, 5–8). This phenomenon may account for the early step in the PrP^Sc^ degradation caused by CPZ and U18666A. In cells persistently infected with prions, PrP^Sc^ exists in organelles in the endocytic-recycling pathway such as early endosomes and ERCs (Figs. 5, 6, and 8, Fig. S3) [20], . PrP^Sc^ also exists in organelles in the endo-lysosomal pathway such as late endosomes and lysosomes (Figs. 7–8) [20], [39], [40]. In the previous study, we reported that PrP^Sc^ is dynamically transported through endocytic compartments by the membrane trafficking machinery of cells [20], and the majority of exogenously introduced PrP^Sc^ is transported to late endosomes/lysosomes via the endo-lysosomal pathway for degradation [25]. These observations suggest that PrP^Sc^ is trafficked via both the endocytic-recycling and endo-lysosomal pathways in persistently infected cells. The decrease in co-localization of PrP^Sc^ with Snx1, EEA1, and Tfn and increase in co-localization of PrP^Sc^ with Lamp1 in CPZ or U18666A-treated cells (Fig. 8) suggest that the treatment induced a transfer of PrP^Sc^ from the endocytic-recycling pathway to the endo-lysosomal pathway. This alteration may increase the opportunity for PrP^Sc^ to be exposed to lysosomal hydrolysis in the endo-lysosomal pathway. The redistribution of PrP^Sc^ to Lamp1-positive vesicles is independent of autophagy because Lamp1-positive vesicles were not co-localized with the autophagosome marker LC3 (Fig. 9).

U18666A is an inhibitor of cholesterol biosynthesis, and it blocks the exit of cholesterol from late endosomes [41], [42]. Gilch *et al.* reported that the accumulation of cholesterol in late endosomes/lysosomes accelerates the degradation of PrP^Sc^ [27]. Recently, Marzo *et al.* reported that tamoxifen, an inhibitor of cholesterol biosynthesis, redistributes PrP^Sc^ to lysosomes along with the accumulation of cholesterol in lysosomes [19]. We also observed the accumulation of cholesterol in Lamp1-positive vesicles after 24 h of CPZ treatment (data not shown). This is similar to the fact that quinacrine, a tricyclic compound, and U18666A redistribute cholesterol from the cell surface to late endosomes/lysosomes in prion-infected Neuro2a cells [14]. Taken together with these observations and the association of PrP^Sc^ with lipid rafts that are cholesterol- and sphingolipid-rich membrane microdomains [43], [44], it is conceivable that the redistribution of PrP^Sc^ to late endosomes/lysosomes upon CPZ or U18666A treatment is associated with the transport of cholesterol-enriched microdomains from the plasma membrane to late endosomes/lysosomes.

CPZ and U18666A required 48 h to reduce PrP-res levels, which was in contrast to the rapid decrease in PrP-res levels induced by mAb 44B1 or PPS within 24 h (Fig. 1). This observation is consistent with a previous report that quinacrine or CPZ requires a few days to decrease PrP^Sc^ levels [45]. The presence of swollen Lamp1-positive vesicles at 6–24 h after CPZ or U18666A treatment and the inhibition of the degradation of LDL in the Lamp1-positive vesicles in CPZ-treated cells suggest temporal impairment of lysosomal activities (Fig. 12), and this impairment may explain the slow degradation of PrP^Sc^. The dysfunction of lysosomal activities was supported by the decrease in lysosomes and increase in late endosomes in Neuro2a cells treated with quinacrine or U18666A [14] or the inhibition of lysosomal hydrolase maturation by U18666A [46]. Nevertheless, the levels of PrP-res and PrP^Sc^ in Lamp1-positive vesicles decreased after 48 h of treatment, at which point swollen Lamp1-positive vesicles disappeared (Figs. 1 and 10), suggesting that PrP^Sc^ was degraded in Lamp1-positive vesicles after lysosomal hydrolysis was restored.

In contrast to the redistribution and relatively slow degradation of PrP^Sc^ induced by CPZ or U18666A treatment, the amount of PrP-res was decreased without an apparent redistribution of PrP^Sc^ within 24 h of treatment with mAb 44B1 or PPS (Figs. 1–3 and 8) that did not appear to affect lysosomal activities (Fig. 12). These data suggest that once PrP^Sc^ formation is inhibited, PrP^Sc^ will be rapidly degraded even in the absence of the transfer of PrP^Sc^ from the endocytic-recycling pathway to the endo-lysosomal pathway. This fact implies that a considerable portion of PrP^Sc^ generated in persistently infected cells may be constantly degraded even in the steady state. Alternatively, the degradation of PrP^Sc^ may occur in both lysosomes and organelles in the endocytic-recycling pathway.

Although we demonstrated that CPZ and U18666A induce the transfer of PrP^Sc^ to late endosomes/lysosomes to be degraded possibly in lysosomes, the alteration of the membrane trafficking machinery and induction of lysosomal dysfunction, with effects below an apparent toxic level with a short exposure, will be disadvantageous for therapeutics. For instance, chronic exposure of primary cultured neurons to U18666A induces intracellular cholesterol accumulation and causes apoptotic cell death, which is similarly observed in cell culture models of Niemann–Pick disease type C [47], a lysosomal storage disorder in CNS [48]. In prion-infected Neuro2a cells, CPZ and U18666A had a narrow effective range compared with that of mAb 44B1 and PPS (data not shown). Considering that therapy for prion diseases requires long-term administration, anti-prion compounds that could interfere with PrP^Sc^ formation and/or enhance its degradation while having little effects on cell metabolism are ideal.

In this study, we could classify the four anti-prion compounds into two groups; one group inhibited PrP^Sc^ formation by influencing PrP^C^ metabolism, and the other induced PrP^Sc^ degradation by altering cell membrane metabolism. To develop more effective but less cytotoxic therapeutics, further analysis using prion-infected cells is required to identify the intracellular event actually involved in PrP^Sc^ clearance induced by any anti-prion compound. Detailed analysis of the intracellular dynamics of PrP^C^ and PrP^Sc^ will provide a logical basis for the development of therapeutic agents for prion diseases.

## Supporting Information

Figure S1 **Effect of the anti-prion compounds on cell proliferation.**
(TIF)

Figure S2 **Double-staining of PrP^Sc^ and PrP^C^.**
(TIF)

Figure S3 **Co-localization of PrP^Sc^ or PrP^C^ with transferrin (Tfn).**
(TIF)
