# Supplementary figures and images for: Comparison of the Anti-Prion Mechanism of Four Different Anti-Prion Compounds, Anti-PrP Monoclonal Antibody 44B1, Pentosan Polysulfate, Chlorpromazine, and U18666A, in Prion-Infected Mouse Neuroblastoma Cells
Source: PLoS One. 2014 Sep 2;9(9):e106516. doi: 10.1371/journal.pone.0106516 (PMC4152300; doi:10.1371/journal.pone.0106516)

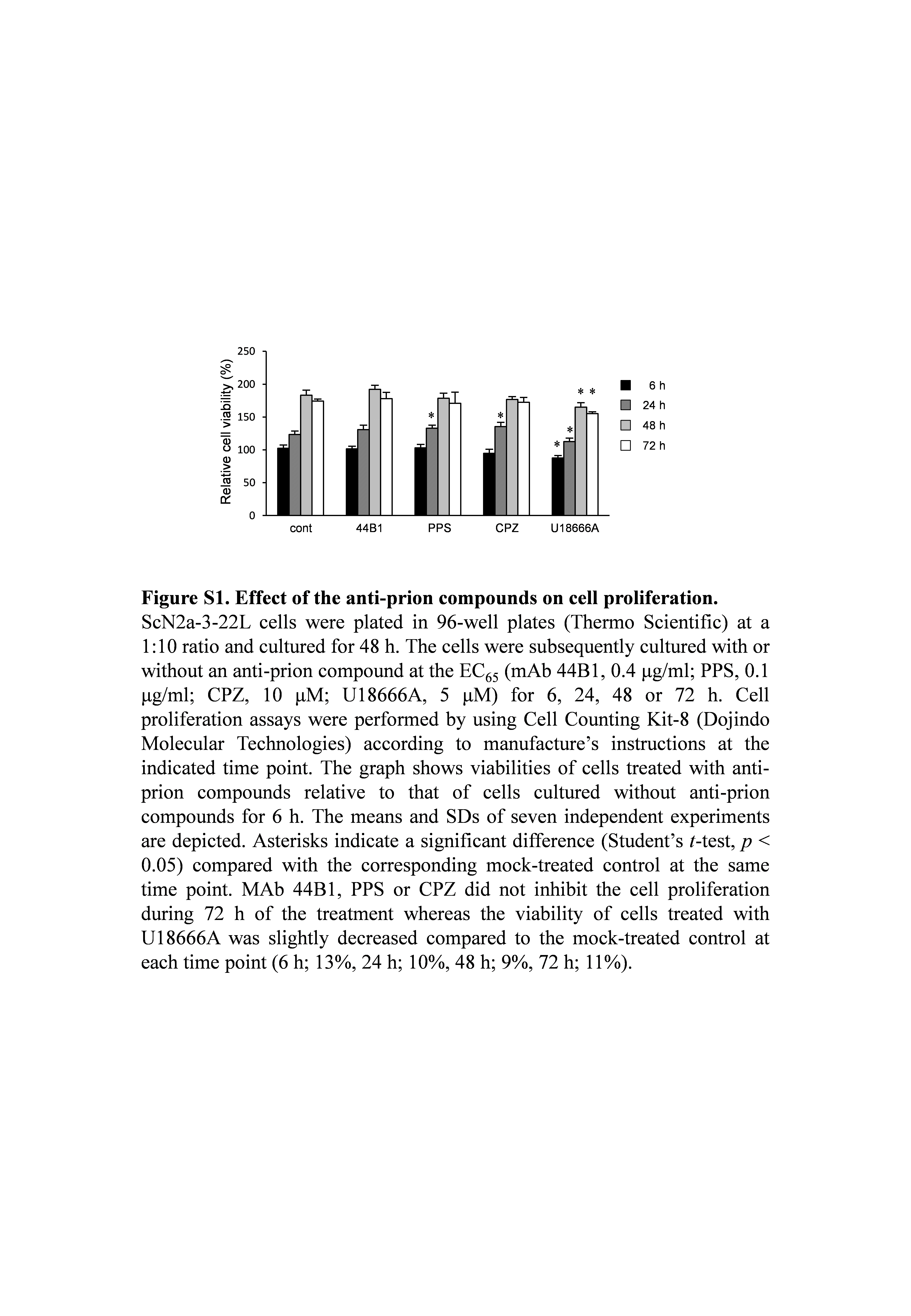

Supplement: Figure S1 — Effect of the anti-prion compounds on cell proliferation. (TIF) [file pone.0106516.s001.tif]

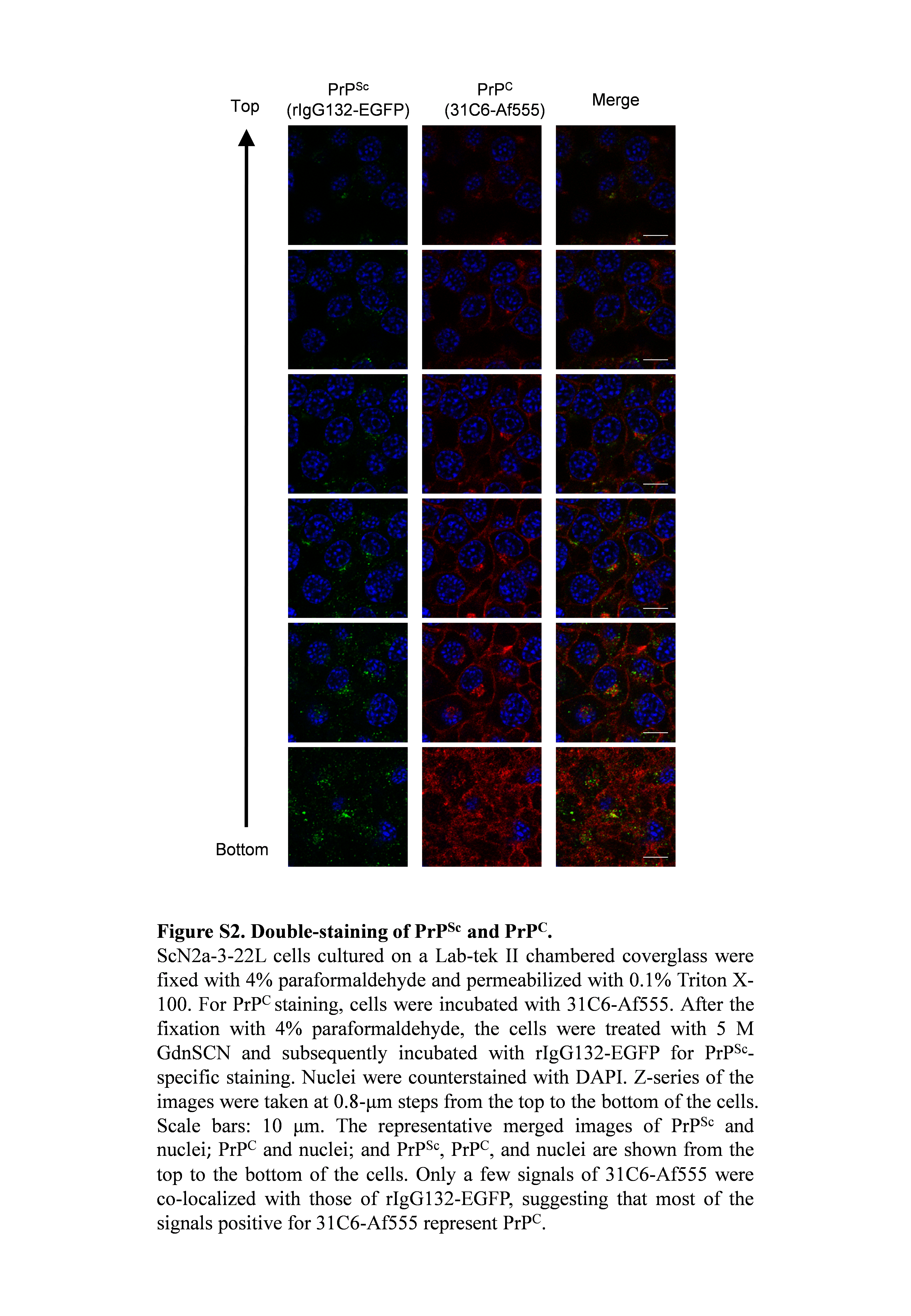

Supplement: Figure S2 — Double-staining of PrPSc and PrPC. (TIF) [file pone.0106516.s002.tif]

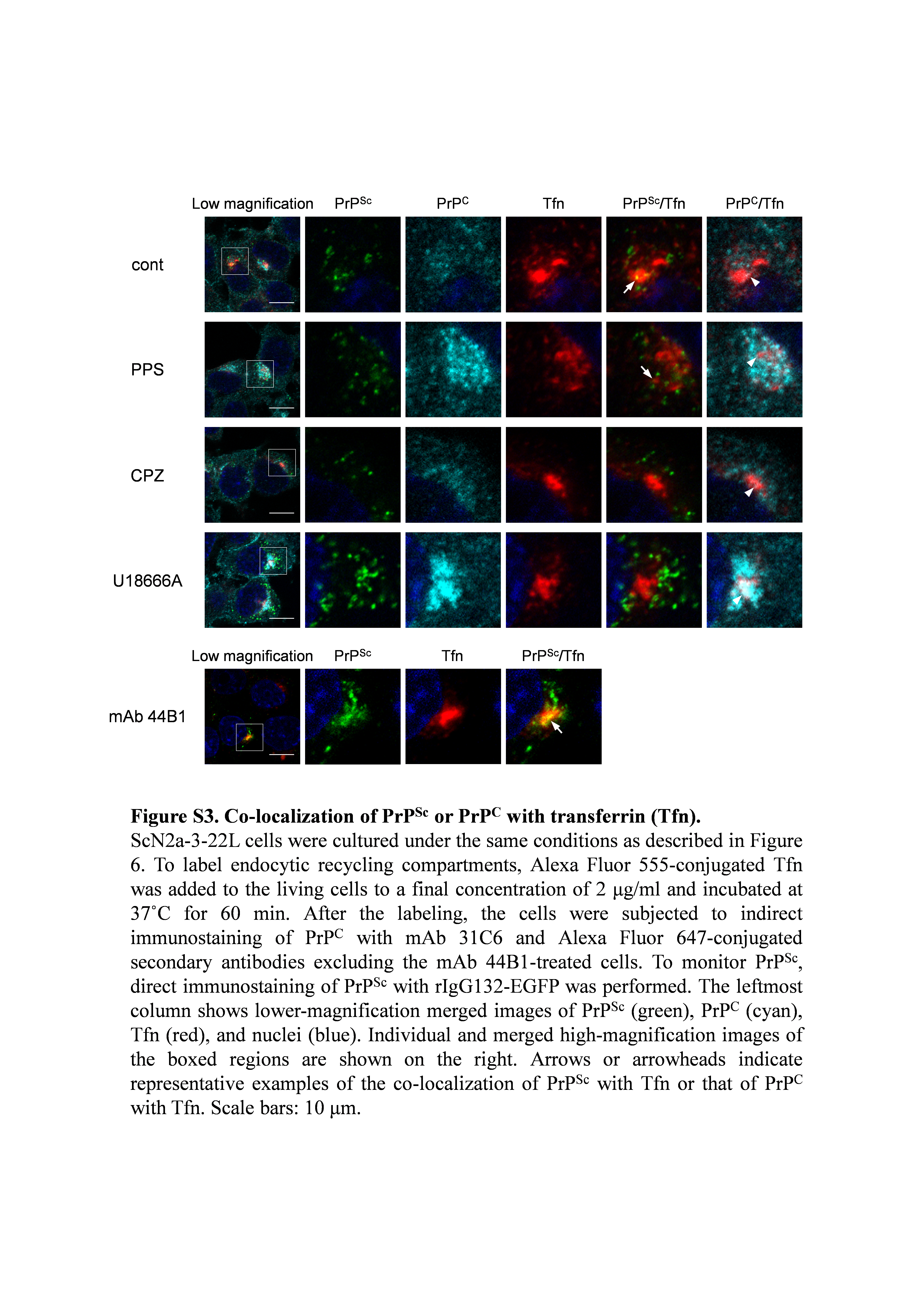

Supplement: Figure S3 — Co-localization of PrPSc or PrPC with transferrin (Tfn). (TIF) [file pone.0106516.s003.tif]
